# Serum retinol-binding protein 4 levels are elevated but do not contribute to insulin resistance in newly diagnosed Chinese hypertensive patients

**DOI:** 10.1186/1758-5996-6-72

**Published:** 2014-06-11

**Authors:** Wuquan Deng, Yuping Zhang, Yanling Zheng, Youzhao Jiang, Qinan Wu, Ziwen Liang, Gangyi Yang, Bing Chen

**Affiliations:** 1 Department of Endocrinology and Metabolism, Southwest Hospital, Third Military Medical University, Chongqing 400038, China; 2 Department of Endocrinology, the Second Affiliated Hospital, Chongqing Medical University, Chongqing 400010, China

**Keywords:** Retinol-binding protein 4, Insulin resistance, Obesity, Essential hypertension

## Abstract

**Background:**

Insulin resistance (IR) is closely correlated with cardiovascular disease (CVD). Retinol-binding protein 4 (RBP4) is a novel adipokine that modulates the action of insulin in various diseases. This study addressed the relationship between RBP4 and IR in newly diagnosed essential hypertension.

**Methods:**

Serum RBP4, anthropometric and metabolic parameters were determined in 267 newly diagnosed essential hypertensive patients not taking antihypertensive medications. The patients along with 64 control (NC) normotensive and lean subjects paired by age and sex were divided into two groups depending on body mass index (BMI), hypertension with obesity (HPO) and hypertension without obesity (HP).

**Results:**

A striking difference was observed in RBP4 levels between the HP and NC groups. Significantly higher levels were noted in the HP group compared with the NC group; slightly, but not significantly, lower levels were observed in the HPO group compared with the HP group. After adjusting for BMI, WC and WHR, a modestly linear relationship was observed between RBP4 levels and SBP (r = 0.377; p = 0.00), DBP (r = 0.288; p = 0.00) and HOMA-β(r = 0.121; p = 0.028). Multiple stepwise regression analysis showed that SBP, WHR and drinking were independently related with serum RBP4 levels.

**Conclusions:**

The results of this study indicated that RBP4 levels were increased in naive hypertensive patients; however, no differences were observed in obese or non-obese hypertensive subjects. Our data suggest for the first time that RBP4 levels are significantly increased but do not contribute to the development of IR in newly diagnosed hypertensive Chinese patients.

## Introduction

Insulin resistance (IR) is one of the major characteristics of cardiovascular disease (CVD). Abundant clinical and epidemiologic evidence demonstrates a close relationship between IR and hypertension [1]. Recent studies have demonstrated that adipocytokines play important roles in the pathogenesis of obesity and hypertension. Retinol-binding protein 4 (RBP4) is a novel adipokine secreted by adipose tissue and the liver that contributes to IR in animal models [2,3]. However, data in humans have been controversial; serum RBP4 levels are reportedly elevated with IR in patients with obesity [2,4] and CVD [5,6], but other authors have failed to confirm these findings [7-10]. In addition, antihypertensive medications were not excluded [10] in previous research despite the fact that angiotensin receptor blockers (ARBs) and calcium-channel blockers (CCBs) can influence serum RBP4 levels [11,12] and IR [13-15]. Previous studies have revealed that RBP4 is closely correlated with atherosclerosis, coronary disease and earlier CVD [5,16]. To date, no studies have evaluated the relationship of RBP4 with IR in largely drug-naive, newly diagnosed hypertensive subjects and normotensive matched individuals. Therefore, in this study, we sought to determine the associations among RBP4 and metabolic indices and IR in newly diagnosed, untreated hypertension patients.

## Subjects and methods

### Study population

We consecutively selected 1400 subjects from the general population who had undergone medical check-ups at the Southwest Hospital Medical Center at the Third Military Medical University from October 2012 to June 2013. After excluding 1069 of the 1400 subjects, a total of 331 subjects were enrolled in our study. In total, 127 newly diagnosed hypertension subjects without obesity (84 men, 43 women, 48.2 ± 8.6 years old, HP group), 140 newly diagnosed patients with hypertension and obesity (97 men, 43 women, 47.7 ± 8.8 years old, HPO group) and 64 normal control subjects (44 men, 20 women, 46.9 ± 7.6 years old, NC group) participated in the study. The diagnosis of hypertension was based on the World Health Organization criteria (1999) [17]. These subjects exhibited stable body weight for at least 3 months prior to testing. The subjects enrolled in the analysis met the following inclusion criteria: (1) age 20 years old or older and younger than 65 years old; (2) fasting blood glucose (FBG) < 6.1 mmol/L and without diabetes or thyroid disease; (3) not taking antihypertensive, antidiabetic or lipid-lowering medications; (4) no clinical symptoms or signs of infection; no liver, kidney or heart disorders or other critical diseases; and no fractures, osteoporosis or tumors; (5) no target organ damage; and (6) not pregnant or lactating. This study was approved by the ethics committee of the Third Military Medical University, and all of the subjects provided written informed consent. Medical histories, including smoking and drinking habits, were obtained from the subjects.

### Anthropometric and blood pressure measurements and definitions

All of the participants were required to fast overnight (8–10 h) before the physical examination. Anthropometric parameters were recorded followed by blood pressure measurements. Then, medical examinations and blood collections were conducted. Blood pressure was measured three times, each 2 min apart on the upper arm using a mercury sphygmomanometer (YUYUE Medical Equipment & Supply CO., LTD., Jiangsu province, China) in a seated, resting position after 10 min of rest; the mean of the three measurements was used for the statistical analysis. Anthropometric parameters, including weight, height and hip and waist circumference (WC), were measured using the International Collaborative Study on Hypertension in Blacks (ICSHIB) standardized protocol [18]. Body mass index (BMI) was calculated as body weight in kilograms divided by the square of height (m^2^). The waist-to-hip ratio (WHR) was calculated as the waist to hip circumference.

We defined current alcohol consumption as more than 1 drink of any type per month and not currently drinking as less than 1 drink of any type per month [19]. Smokers were defined as having smoked more than 100 cigarettes in one’s lifetime and having smoked at least one cigarette daily for 6 months by the time of the interview [20]. Obesity was defined as body mass index (BMI) ≥ 25 kg/m^2^ as suggested by the WHO Western Pacific Regional Office [21]. Hypertension was defined as a systolic blood pressure (SBP) ≥ 140 mmHg and/or a diastolic blood pressure (DBP) ≥ 90 mmHg [17].

### Experimental procedures and serum samples

Overnight fasting blood samples were collected in tubes containing liquid EDTA; the samples were centrifuged at -4°C and maintained at -80°C until assayed. A certified laboratory assessed metabolic parameters, including fasting blood glucose (FBG), blood uric acid (UA), total cholesterol (TC), triglycerides (TG), low-density lipoprotein C (LDL-C), high-density lipoprotein C (HDL-C) and fasting insulin (FINS). FBG was assayed using the glucose oxidase method. UA, TC, TG, LDL-C and HDL-C concentrations were determined enzymatically. Serum RBP4 levels were evaluated using a commercially available enzyme-linked immunosorbent assay (ELISA; R&D Systems, Inc., Minneapolis, MN, USA). The intra-assay coefficients of RBP4 were less than 10%, and the interassay coefficients were less than 15%. The linear ranges of the assays were 1.0 to 300 μg/ml for RBP4.

The homeostasis model assessment of IR (HOMA-IR) as an indicator of IR and the homeostasis model assessment of β-cell insulin secretion (HOMA-β) were calculated from FINS and FBG levels using the following equations: HOMA-IR = FINS (μU/mL) × FBG (mmol/L)/22.5; and HOMA-β = (20 × FINS [μU/ml])/(FBG [mmol/L - 3.5]) [22].

### Statistical analysis

The experimental data were analyzed using SPSS software (SPSS, Chicago, IL, USA), version 17.0. Continuous variables are presented as means ± standard deviations, and categorical variables are presented as absolute and relative frequencies (%). Before the statistical analysis, the data were subjected to normal distribution analysis using the Kolmogorov-Smirnov test. Non-normally distributed data (TG, FINS, HOMA-IR and HOMA-β) were converted by logarithmic transformation. Categorical variables were compared using the chi-square test. The differences among groups were tested using analysis of variance (ANOVA). In addition, a post-hoc least-significant difference (LSD) test was used for equal variances assumed variables, and Dunnett’s test was used for equal variances not assumed variables. Interrelationships between variables were analyzed by Pearson correlation analysis. Partial correlation analysis was performed to control for body fat parameters. Multiple linear regression analysis was performed using stepwise linear regression to correct the effects of the covariates and to test independent factors. A p-value < 0.05 was considered to be statistically significant for all of the analyses.

## Results

The three groups of subjects were well matched for age and sex, and all of the subjects with hypertension were naive to antihypertensive therapy. The clinical characteristics of the study subjects are presented in Table 1. TC and LDL-C did not differ among the three groups. The HPO group displayed higher BMI, WC, WHR, SBP, DBP, UA, TG, FINS, HOMA-IR, HOMA-β, drinking (all p < 0.01) and FBG (p < 0.05) but lower HDL-C (p < 0.01) than the NC group. The HPO group had higher BMI, WC, WHR, UA, TG, FBG, FINS, HOMA-IR, drinking (all p < 0.01) and SBP (p < 0.05) but lower HDL-C (p < 0.01) than the HP group. The HP group had higher SBP, DBP, TG, FINS, HOMA-IR, HOMA-β (p < 0.01), WC, WHR and UA (p < 0.05) than the NC group.

**Table 1:** Clinical characteristics of study subjects

	**NC (n = 64)**	**HP (n = 127)**	**HPO (n = 140)**	**p-value**
Age (yr)	46.9 ± 7.6	48.2 ± 8.6	47.7 ± 8.8	0.602*
Sex (M/F)	97/43	84/43	44/20	0.852^#^
BMI (kg/m^2^)	22.2 ± 1.3	22.5 ± 1.7	27.8 ± 1.9^ab^	0.000*
WC (cm)	81.1 ± 5.4	83.0 ± 6.7^c^	94.7 ± 6.0^ab^	0.000*
WHR	0.88 ± 0.05	0.90 ± 0.05^c^	0.94 ± 0.05^ab^	0.000*
SBP (mmHg)	116.5 ± 11.4	151.7 ± 11.7^a^	155.8 ± 14.7^ad^	0.000*
DBP (mmHg)	74.5 ± 7.7	97.0 ± 9.4^a^	98.8 ± 11.4^a^	0.000*
UA (µmol/l)	294.2 ± 67.7	323.0 ± 78.8^c^	384.8 ± 91.4^ab^	0.000*
TG (mmol/l)	1.39 ± 0.65	1.93 ± 1.07^a^	2.43 ± 1.27^ab^	0.000*
TC (mmol/l)	5.31 ± 0.95	5.38 ± 0.92	5.40 ± 0.97	0.658*
LDL-C (mmol/l)	2.53 ± 0.60	2.70 ± 0.58	2.72 ± 0.63	0.176*
HDL-C (mmol/l)	1.60 ± 0.28	1.53 ± 0.33	1.42 ± 0.33^ab^	0.001*
FBG (mmol/l)	5.45 ± 0.42	5.42 ± 0.57	5.64 ± 0.56^bc^	0.003*
FINS (pmol/l)	8.4 ± 3.90	10.7 ± 3.57^a^	14.6 ± 5.40^ab^	0.000*
HOMA-IR	2.06 ± 1.06	2.60 ± 1.00^a^	3.66 ± 1.40^ab^	0.000*
HOMA-β	87.4 ± 32.7	119.7 ± 48.8^a^	149.5 ± 83.9^a^	0.000*
Smoking (%)	32.8%	37.0%	33.6%	0.788^#^
Drinking (%)	26.6%	34.7%	51.4%^ab^	0.001^#^

Data represent means ± SD or frequency (percentage).

TG, FINS and HOMA-IR were log transformed before analysis.

*Analysis of variance; ^#^ chi-squared test.

^a^p < 0.01 compared with NC.

^b^p < 0.01 compared with HP.

^c^p < 0.05 compared with NC.

^d^p < 0.05 compared with HP.

NC, normal control subject; HP, hypertension with obesity; HPO, HP with obesity; BMI, body mass index; WC, Waist circumference; WHR, waist-to-hip ratio; SBP, systolic blood pressure; DBP, diastolic blood pressure; WHR, waist hip ratio; UA, uric acid; TG, triglyceride; TC, total cholesterol; LDL-C, low-density lipoprotein cholesterol; HDL-C, high-density lipoprotein cholesterol; FBG, fasting blood glucose; FINS, fasting plasma insulin; HOMA-IR, HOMA-insulin resistance index; HOMA-β, HOMA β cell function index.

**Figure 1:**
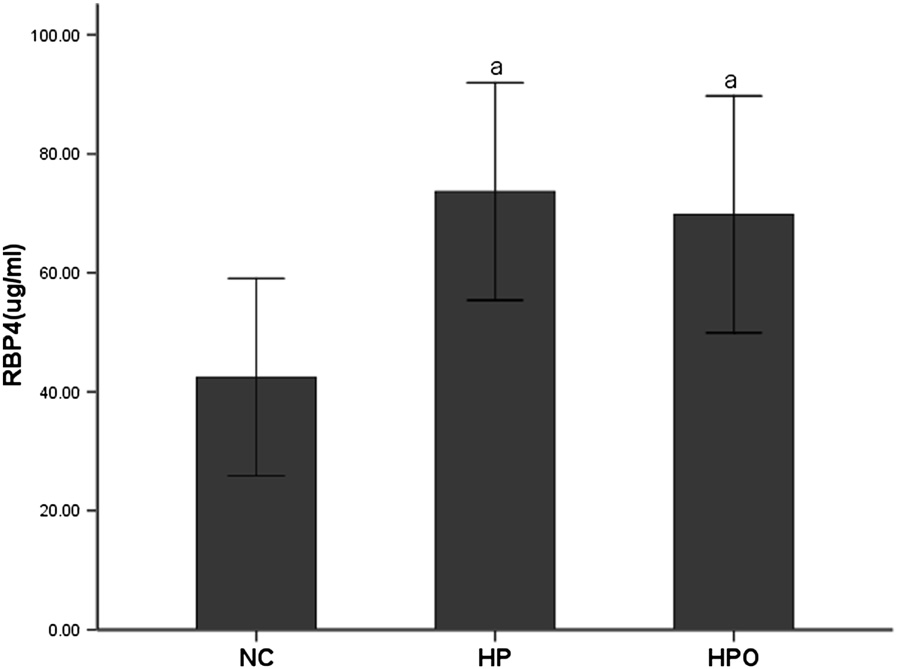
**Serum RBP4 levels in NC, HP and HPO groups. **^a^p < 0.01 compared with NC.

As presented in Figure 1, serum RBP4 levels were significantly increased in the hypertensive subjects (HP and HPO groups) compared with the NC group (73.6 ± 18.3 μg/ml, 69.8 ± 19.9 μg/ml versus 42.4 ± 16.6 μg/ml, both p < 0.01). However, a decreasing trend in serum RBP4 concentrations was observed in the HPO group compared with the HP group (69.8 ± 19.9 μg/ml versus 73.6 ± 18.3 μg/ml, p = 0.093), but the trend was not statistically significant. Bivariate correlation analyses were performed to assess relationships between RBP4 levels and anthropometric or metabolic parameters. RBP4 was positively correlated with BMI (r = 0.108; p = 0.049), SBP (r = 0.381; p = 0.00), DBP (r = 0.296; p = 0.00), TG (r = 0.127; p = 0.021), FINS (r = 0.116; p = 0.035), HOMA-IR (r = 0.121; p = 0.027), and HOMA-β (r = 0.153; p = 0.005). After adjusting for BMI, WC and WHR, positive correlations between RBP4 and SBP (r = 0.377; p = 0.00), DBP (r = 0.288; p = 0.00) and HOMA-β (r = 0.121; p = 0.028) were still evident, but the correlations between RBP4 and TG (r = 0.106; p = 0.055), FINS (r = 0.068; p = 0.219) and HOMA-IR (r = 0.075; p = 0.177) disappeared. Using age, sex, BMI, WC, WHR, SBP, DBP, UA, TC, TG, LDL-C, HDL-C, FBG, FINS, HOMA-IR and HOMA-β as independent variables, multiple stepwise regression analysis revealed that SBP, WHR and drinking were independently related to RBP4 levels (β = 0.397, -0.175, 0.181, all p < 0.05). The multiple regression equation was as follows: Y_RBP4_ = 59.27 + 0.45X_SBP_ ‒ 68.06X_WHR_ + 8.01X_Drinking_ (Table 2).

**Table 2:** Linear and multiple regression analysis of anthropometric and metabolic parameters associated with RBP4 in subjects studied

	**Simple**		**Multiple**	
**Variable**	**Estimate**	**p-value**	**Estimate**	**p-value**
BMI (kg/m^2^)	_	_	_	_
WC (cm)	_	_	_	_
WHR	_	_	-0.175	0.003
SBP (mmHg)	0.377	0.000	0.397	0.000
DBP (mmHg)	0.288	0.000	_	_
UA (µmol/l)	0.014	0.796	_	_
TG (mmol/l)	0.106	0.055	_	_
TC (mmol/l)	0.014	0.802	_	_
LDL-C mmol/l)	0.031	0.612	_	_
HDL-C (mmol/l)	0.067	0.268	_	_
FBG (mmol/l)	0.045	0.416	_	_
FINS (pmol/l)	0.068	0.219	_	_
HOMA-IR	0.075	0.177	_	_
HOMA-β	0.121	0.028	_	_
Drinking	_	_	0.181	0.002

In multiple regression analyses, p-values were calculated after adjusting for BMI, WC and WHR.

TG, FINS, HOMA-IR and HOMA-β were log transformed before analysis.

## Discussion

### Associations among RBP4 and markers of IR and metabolic indices

Previous studies have yielded conflicting results regarding the relationship between RBP4 and IR [23,24]. Animal models and some human research have reported that RBP4 contributes to IR [2-4]. However, other relationships have been inconsistently demonstrated. Von Eynatten et al. revealed no significant correlations between RBP4 levels and BMI or HOMA-IR in 143 patients with coronary artery disease [25]. Similarly, Shim et al. found that RBP4 was not associated with IR or metabolic indices in 308 non-diabetic hypertensive patients, but this report did not exclude the influence of antihypertensive medications [10]. Therefore, it is worth exploring the clinical significance of RBP4 in newly diagnosed, untreated hypertension patients.

In this study, we detected RBP4 levels in humans and investigated the relationships between RBP4 levels and relevant factors. Given that adipokine expression may be affected by sex hormones, a study reported that increases in RBP4 levels following blood pressure increases exclusively occurs in women and not men [9]. We compared serum RBP4 levels between genders, but no sex difference was observed. We found that HOMA-IR was significantly elevated in the HP group compared with NC group and rapidly increased along with obesity in the HPO group. RBP4 was positively correlated with SBP, DBP, TG, FINS, HOMA-IR and HOMA-β; however, the correlation between RBP4 and TG, FINS or HOMA-IR disappeared after controlling for body fat parameters.

In present study, we found that serum RBP4 levels were significantly increased in the hypertensive subjects but do not contribute to IR after excluding the confounders of obesity. These results are consistent with those identified in previous reports [10,23] but inconsistent with the original report of the contribution of RBP4 to IR [2-4]. RBP4 levels were slightly positively associated with insulin secretion (HOMA-β), consistent with Li L et al.’s previous report [26]. Insulin sensitivity and serum insulin concentrations are mutually related, and Burattini et al. [27] reported that insulin clearance was decreased in hypertensive patients but not affected by metabolic syndrome. Decreased insulin clearance is another regulatory mechanism, in addition to increased insulin secretion, that compensates for insulin resistance. Interestingly, alcohol consumption independently influences RBP4, and we hypothesize that chronic ethanol consumption disrupts glucose homeostasis and is associated with the development of IR. In addition, hypertension associated with IR is characterized by an infiltration of macrophages into adipose tissues as well as changes in the expression of adipocytokines.

### Relationship between RBP4 and hypertension

Previous research indicates that RBP4 is a possible predictor of CVD [28,29]. Alkharfy et al. suggested that RBP4 might serve as an independent predictor of CVD in 284 women with good health, obesity, diabetes, obesity and diabetes or CVD [28]. In our results, we found that RBP4 levels were higher in hypertension patients than in normotensive, normal-weight patients. RBP4 was strongly correlated with SBP and DBP, and the former was an independently correlated factor of RBP4. Similar findings have been reported previously. Solini et al. reported that RBP4 levels were significantly increased in 35 untreated essential hypertensive women compared with in 35 normotensive lean women, and RBP4 levels were strongly and directly correlated with SBP and DBP values in all of their subjects [29].

## Conclusions

Our study population, which contained a sufficient number of subjects, was strictly matched for age and sex, thus likely excluding the influence of related metabolic alterations on RBP4 levels. In summary, for the first time, we have demonstrated that serum RBP4 levels were significantly increased, but RBP4 did not appear to be a valuable marker for the identification of IR in newly diagnosed hypertensive Chinese patients.

There were also some limitations to our study. Our study subjects consisted of men and women of Chinese ethnicity; hence, the generalizability of our study to other ethnicities is unknown [30,31]. Although several potential risk factors for CVD were included in our analysis, the role of some unknown or unmeasured confounders cannot be excluded. We did not perform oral glucose tolerance tests or glucose clamp studies to determine insulin resistance because of condition limitations. This finding should be investigated further in experimental follow-up studies.

## Abbreviations

IR: Insulin resistance; CVD: Cardiovascular disease; RBP4: Retinol-binding protein 4; BMI: Body mass index; WC: Waist circumference; WHR: Waist-to-hip ratio; SBP: Systolic blood pressure; DBP: Diastolic blood pressure; FBG: Fasting blood glucose; FINS: Fasting insulin; UA: Uric acid; TG: Triglycerides; HDL-C: High-density lipoprotein C; LDL-C: Low-density lipoprotein C; HOMA-IR: Homeostasis model assessment of IR; HOMA-β: Homeostasis model assessment of β-cell insulin secretion.

## Competing interests

The authors declare that they have no competing interests.

## Authors’ contributions

DWQ and ZYP participated in the data collection, checked the data, performed the statistical analysis and wrote the manuscript; ZYL, JYZ, YGY, WQN and LZW contributed to the discussion; CB participated in the design of this study and edited the manuscript. All of the authors have read and approved the final manuscript.
